# Short report: Prison-reported rates of autism in female prisons in England

**DOI:** 10.1177/13623613241275477

**Published:** 2024-09-19

**Authors:** Katy-Louise Payne, Emma Gooding

**Affiliations:** University of Northampton, UK

**Keywords:** autism, autism spectrum disorder, crime, female, offending

## Abstract

Research suggests that whilst not more likely to offend, autistic people are overrepresented within the Criminal Justice System. To date, prevalence estimates are available only for male autistic offenders. Thus, this research aimed to provide the prison-reported rate of autistic female prisoners currently residing in the female prison estate in England. All 12 of the female prisons in England were contacted to request the following information: (1) the total number of autistic females; and (2) the total female prison population. Findings show an English female prison-reported rate of autism of 4.78% indicating that autism is approximately 13.7 times higher in female prisons compared to the general population. To the authors’ knowledge, this is the first research article to focus exclusively on female autistic offenders. Further research is needed to understand the risk factors for offending, offender characteristics, offence types/patterns and autistic experience of the Criminal Justice System for female autistic offenders.

**Lay abstract:**

**What is already known about the topic?** Autistic people are reported to offend at lower or comparable rates to non-autistic people. However, autism is overrepresented within male prisons due to a number of suggested reasons including an increased chance of being caught and a lack of autistic sensitive interventions which lead to longer stays in prison. To the authors’ knowledge, no papers currently exist focussing on autistic females in prison. **What does this paper add?** To the authors’ knowledge, this is the first piece of research to solely include autistic females in prison settings. This research provides an estimate of how many autistic people are in female prisons. All 12 female prisons in England were contacted. Data provided indicate a prison-reported female autism rate of 4.78%. This prison-reported female autism rate is 13.7 times higher than the prevalence of autistic females in the general population. **Implications for practice research or policy?** Autism is overrepresented in female prisons; however, to the authors’ knowledge no current research exists on this group to understand their needs or experience. Autistic females often have differing requirements to males and the lack of research highlights the need for future research to investigate areas such as factors which increase the risk of offending, offences typically committed and the female autistic experience of the Criminal Justice System.

## Introduction

### Female autism prevalence

In England, the reported combined male and female autism prevalence is approximately 0.78% (O’Nions et al., 2023) with variation in prevalence rates observed between different geographical areas (Roman-Urrestarazu et al., 2021). A higher prevalence of autism is reported in males compared to females in the non-offending population with current estimates finding the male to female ratio to be approximately 3:1 (Loomes et al., 2017). The female autism prevalence in England is reported to be approximately 0.35% compared to 1.22% in males (O’Nions et al., 2023). There are a number of proposed explanations for the autistic gender imbalance including the idea that there is a subtle yet distinct female phenotype of autism that current autism diagnostic tools cannot effectively capture (Lai et al., 2015; Van Wijngaarden-Cremers et al., 2014) or that females are better at masking their difficulties (Baldwin & Costley, 2016; Bargiela et al., 2016; Cridland et al., 2014). In addition, female individuals attempting to obtain an autism diagnosis report significantly more barriers to diagnosis (e.g. due to camouflaging behaviours, fearing not being believed) and often experience later or missed diagnosis (Cook et al., 2024; Lewis, 2017).

### Female autistic presentation and experience

Despite the lower reported autism prevalence in females in the general population, research suggests that autistic females may be at greater risk of negative comorbidities than autistic males including internalising symptoms (e.g. low self-esteem) and presenting with greater emotional disorders (e.g. anxiety, depression; Cook et al., 2018; May et al., 2014; Schuck et al., 2019). Autistic females often report loneliness and isolation which often results in bullying or ostracism (Cook et al., 2018). This leads to autistic females developing coping strategies to fit in to their social environment (e.g. imitating peers, masking autistic traits; Schuck et al., 2019). While some autistic females may be able to effectively fit into their social environment, there are many associated negative consequences to camouflaging their autism/autistic traits. These include late diagnosis resulting in delayed access to person centred care, treatment, intervention (Cage & Troxell-Whitman, 2019; Head et al., 2014; Hull et al., 2017, 2019; Lai et al., 2017) and negative emotional responses (e.g. anxiety, depression, loss of identity, low self-esteem, self-injurious behaviour, suicidal thoughts; Cook et al., 2018; Hull et al., 2017, 2019; Lai et al., 2017).

### Autism in the criminal justice system

To the authors’ knowledge, there is no female autistic prison prevalence data published. However, research suggests that the prevalence of autism within male prison settings is approximately four times higher than the general population (Fazio et al., 2012) with autistic offenders frequently reporting a negative experience within the Criminal Justice System in England and Wales (CJS; Allen et al., 2008; Crane et al., 2016). Despite the increased prison prevalence rate, a recent systematic review suggests that autistic people offend at a lower or comparable rate to the general population (Chester et al., 2022). It is suggested when looking at studies that measure the number of autistic people within the CJS that autistic people are overrepresented (Chester et al., 2022). A number of reasons for the overrepresentation are suggested, including (1) being more likely to get caught; (2) being more likely to have difficulty advocating for rights; and (3) a lack of autistic sensitive rehabilitation programmes and risk assessments which may lead to longer forensic stays (Chester et al., 2022).

### Prisons in England

Within England, there are 12 prisons that accommodate female offenders (Beard, 2023). Prisons in England are either publicly run by His Majesty’s Prison and Probation Service (HMPPS) or privately run with HMPPS managing the contracts (Beard, 2023). Currently, there are two privately run female prisons and 10 publicly run prisons. Female prisons are categorised as either open (i.e. minimal perimeter and physical security) or closed (i.e. secure perimeter and range of internal security measures; HM Inspectorate of Prisons, 2023; Ministry of Justice, 2024a). Currently, there are two open prisons. If an offender is sent to prison, they are assigned to the lowest security category to manage their risk (Ministry of Justice, 2021).

Currently in the England, there are approximately 3664 females within the prison estate (Ministry of Justice, 2024b) with evidence highlighting that the general female offender population have a higher prevalence of need (e.g. mental health difficulties, Ministry of Justice, 2018; Tyler et al., 2019). Jain (2023) highlights the importance of identifying the prevalence of a diagnosis to enable planning (e.g. resources, facilities and interventions). However, the number of autistic females currently residing in prisons in England is unknown. Thus, it could be hypothesised that this lack of knowledge of female autism prevalence in prisons negatively impacts planning for treatment, rehabilitation and ultimately outcomes for autistic female offenders (Jain, 2023).

### The present study

Research highlights increased needs and comorbidities for both the autistic female population and the female offender population (e.g. Cook et al., 2018; May et al., 2014; Ministry of Justice, 2018; Schuck et al., 2019; Tyler et al., 2019). However, to the best of the researcher’s knowledge, no research papers have specifically focused on the intersectionality of autistic female offenders. This study seeks to address this. The study aims to provide the prison-reported rate of autistic female prisoners in England.

## Methods

### Procedures

Within England and Wales, female offenders account for approximately 4% of the prison estate (*n* = 3664; Ministry of Justice, 2024b) located within 12 prisons (Beard, 2023). There are no female prisons in Wales. All of the female prison establishments in England were contacted and the following data obtained and recorded: (1) the total number of autistic females and (2) the total female prison population. Prisons were not explicitly directed to provide professionally diagnosed and/or self-identified autistic offenders. The rationale for not stipulating professionally or self-diagnosed was to increase inclusivity (Overton et al., 2023). Previous research highlighted the barriers to diagnosis the females face, which often lead to later or missed autism diagnosis (Cook et al., 2024; Lewis, 2017).

Data were retrieved between 2 February 2023 and 22 March 2023 from computer systems/records stored within each prison rather than a nationwide collection of data. This is because currently there is no defined way to record autism diagnosis on a national basis. The 12 prison establishments contacted are detailed in Table 1 in addition to further information about each establishment (e.g. prison size, security classification) to provide context for readers outside England. Ethical approval for this study was granted from the University of Northampton Research Ethics Committee (reference number: FHSHEA000307).

**Table 1.** Overview of female prison characteristics in England.

Prison	Certified Normal Accommodation^a^	Security Classification	Public or Privately Run
Prison 1	128	Open	Public
Prison 2	505	Closed	Private
Prison 3	356	Closed	Public
Prison 4	340	Closed	Public
Prison 5	137	Open	Public
Prison 6	389	Closed	Public
Prison 7	286	Closed	Public
Prison 8	361	Closed	Public
Prison 9	341	Closed	Public
Prison 10	360	Closed	Private
Prison 11	266	Closed	Public
Prison 12	469	Closed	Public

aThis is the number of prisoners that the prison can accommodate in a good, decent standard of accommodation (i.e. uncrowded capacity; Ministry of Justice, 2024b)

### Community involvement statement

While no autistic female offenders directly participated in the data collection of this project, two autistic stakeholders provided verbal feedback in developmental stages of the study. Their contributions were instrumental in shaping the research’s aims and underlying rationale, and their insights, drawn from personal experience, informed the study.

## Results

Data were obtained for the 12 prison establishments. The exact numbers of autistic prisoners residing within two prisons were not provided due to concerns data would breach General Data Protection Regulations (GDPR) and/or Data Protection Act (2018). This was specifically due to the prisons reporting five or fewer autistic females and concerns of individual identification with these small numbers.

The overall prison-reported autism rate in the female prison estate in England was 4.78%. Table 2 provides an overview of each prison and their prison-reported autism rate.

**Table 2.** Prison-reported autism rate by prison.

Prison	Autistic Prisoners	Total Prisoners	Prison-reported Autism Rate
Prison 1	0	86	0.00%
Prison 2	20	470	4.26%
Prison 3	17	259	6.56%
Prison 4	8	318	2.52%
Prison 6	11	351	3.13%
Prison 7	7	241	2.90%
Prison 9	10	330	3.03%
Prison 10	12	317	3.79%
Prison 11	11	175	6.29%
Prison 12	45	402	11.19%
Total	141	2949	4.78%

## Discussion

To our knowledge, this study is the first globally to present the number of autistic females currently residing within the female prison estate. It was found that 4.78% of female prisoners were reported to be autistic. This figure is 13.7 times higher than the female autism prevalence in the general population (0.35%; O’Nions et al., 2023). Variations in prison-reported autism rates were observed between prisons ranging from 0% (Prison 1) to 11.19% (Prison 12), which may support previous research reporting geographical differences in autism diagnosis due to varying services, funding, policies and strategies (Roman-Urrestarazu et al., 2021). Overall, this study suggests that autistic females may be at greater risk of being overrepresented within the prison estate.

While we have limited research which includes female autistic offender participants, the male dominated autistic offender research identifies negative experiences within the CJS. Experiences include vulnerability and bullying (Newman et al., 2015; Patterson, 2008) with autistic offenders typically reporting an overall negative experience within the CJS in England and Wales (Allen et al., 2008; Crane et al., 2016). This finding coupled with the heightened risk of negative comorbidities in non-offending autistic females compared to males (Cook et al., 2018; May et al., 2014; Schuck et al., 2019) and imitating peers to try and fit in to the social environment (Schuck et al., 2019) could lead to a hypothesis of increased difficulties and challenges for female autistic offenders within the CJS. However, given the limited female autistic offender research, this needs to be investigated further.

To enable evidence-based practice (i.e. using the best available evidence to develop policy and programmes; World Health Organization, 2021), further research on female autistic offenders is required. Future research should investigate (1) prevention of female autistic offending (e.g. through identification of risk factors for offending, offender characteristics, offence types and offending patterns); (2) female autistic offenders’ needs and presentation while incarcerated to inform intervention(s); and (3) female autistic experience of the CJS.

A limitation of this current research is that within the prison service in England there is no standardised method to record an autism diagnosis and as such the data provided were drawn from computer systems/records within each prison. For this research, it was not possible to identify whether the female autistic offenders had a formal diagnosis or self-identified as autistic. Research suggests that self-identifying adults often do this due to challenges with being referred or formally assessed (Overton et al., 2023), which is common in autistic females (Gesi et al., 2021). Camouflaging (i.e. minimising/masking autistic traits in social settings), misdiagnosis and diagnostic overshadowing are common in autistic females, which may lead to missed formal diagnosis suggesting that autism may be underdiagnosed in females (Hull et al., 2020). Challenges for diagnosis are compounded within forensic settings where additional barriers to accurate autism assessment can occur (e.g. difficulties obtaining developmental histories from parents/caregivers). While some research may question the accuracy of autistic self-report, other research suggests that self-report of experience by autistic adults does not significantly differ to informant-report indicating autism self-identification should be reliable (Sandercock et al., 2020). Diagnostic challenges and autistic self-awareness of experience highlights the importance of including the self-identified autistic population in this current autistic female offender research. It is hoped that the current research will increase awareness of female autistic offenders in prison to highlight some of the possible challenges female autistic offenders may face and be used to help inform both future research and practice.

A second limitation is the reliance on the prisons computer systems/records for obtaining autistic status. It is possible that while some self-identifying autistic people are identified through this system, others may choose not to disclose their self-identifying autistic status. The final limitation is that while this research focussed specifically on those residing in English female prisons we did not ask for gender identity. Future research should investigate gender identity within the female prison estate to inform understanding.

In summary, results indicate that autism appears to be overrepresented within the female prison estate in England. The self-reported autism rate of 4.78% within female prisons suggests that when compared to the general population autism is approximately 13.7 times higher in female prisons in England. This highlights the overrepresentation of autistic females in prisons in England. Despite this finding, coupled with non-offender female research indicating that autistic females may experience more negative comorbidities associated with autism (e.g. emotional disorders including anxiety and depression; Cook et al., 2018; Schuck et al., 2019), increased vulnerability/risk (e.g. through imitating peers to try and fit in to the social environment; Schuck et al., 2019) and barriers to accessing support (Doherty et al., 2022), there is no published research to date focussing on autistic females within the CJS. This highlights a clear gap in the evidence base which future research needs to address.
